# POC device for rapid oral pH determination based on a smartphone platform

**DOI:** 10.1007/s00604-024-06227-1

**Published:** 2024-02-14

**Authors:** Manuel J. Arroyo, Pablo Escobedo, Isidoro Ruiz-García, Alberto J. Palma, Francisco Santoyo, Mariano Ortega-Muñoz, Luis Fermín Capitán-Vallvey, Miguel M. Erenas

**Affiliations:** 1 Department of Analytical Chemistry, ECsens, University of Granada, Campus Fuentenueva, Granada, Spain; 2 ECsens, CITIC-UGR, iMUDS, Department of Electronics and Computer Technology, University of Granada, Granada, Spain; 3 Unit of Excellence in Chemistry Applied to Biomedicine and the Environment of the University of Granada, Granada, Spain; 4 Department of Organic Chemistry, University of Granada, Campus Fuentenueva, Granada, Spain

**Keywords:** Colourimetric sensors, Smartphone color detection, Salivary pH determination, Anthocyanins, Poin-of-Care (POC)

## Abstract

Salivary pH serves as a valuable and useful diagnostic marker for periodontal disease, as it not only plays a critical role in disease prevention but also in its development. Typically, saliva sampling is collected by draining and spitting it into collection tubes or using swabs. In this study, we have developed a Point-of-Care (POC) device for in situ determination of oral pH without the need for complex instruments, relying solely on a smartphone as the detection device. Our system utilizes a non-toxic vegetable colourimetric indicator, immobilized on a chitosan membrane located on a disposable stick, enabling direct sampling within the buccal cavity. An ad hoc designed 3D-printed attachment is used to ensure accurate positioning and alignment of the stick, as well as isolation from external lighting conditions. A custom-developed smartphone application captures and automatically processes the image of the sensing membrane, providing the salivary pH results. After optimizing the cocktail composition, the developed sensors demonstrated the capacity to determine pH within a range of 5.4 to 8.1 with a remarkable precision of 0.6%, achieving a very short analysis time of just 1 min. A stability study conducted on the sensing membranes revealed a lifetime of 50 days. To validate the performance of our analytical device, we compared its results against those obtained from a calibrated pH-meter, using a group of individuals. The device exhibited an average error of 2.4% when compared with the pH-meter results, confirming its reliability and accuracy.

**Graphical abstract:**

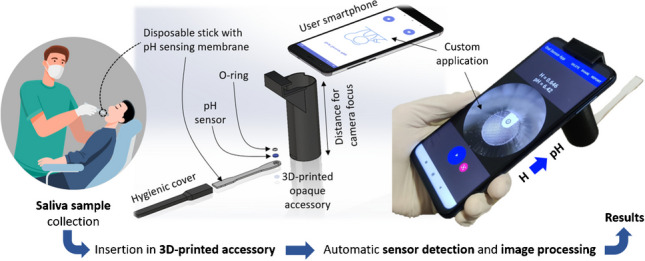

**Supplementary Information:**

The online version contains supplementary material available at 10.1007/s00604-024-06227-1.

## Introduction

The use of saliva as a diagnostic biofluid has witnessed a significant increase in recent years [1]. This can be attributed to the identification and validation of new biomarkers, as well as advancements in test accuracy, sensitivity, and precision, which have enabled the development of novel non-invasive and cost-effective devices that enhance bedside patient monitoring and enable early treatment. Saliva is widely acknowledged as one of the most suitable biological samples for the exploration of potential biomarkers associated with systemic diseases, such as cancer, diabetes, cardiovascular diseases, and autoimmune disorders [2, 3]. The diverse range of biomolecules present in saliva, including enzymes, hormones, antibodies, and RNA and DNA fragments [4, 5], can be effectively monitored and correlated to the patient’s health condition. On the other hand, the salivary proteome consists of about 2,000 characteristic proteins and peptides that can be used to diagnose a multitude of pathologies [6]. Furthermore, saliva has been employed as a detection medium for ultrasensitive in-situ immunoassay, serving as a diagnostic tool for Severe Acute Respiratory Syndrome Coronavirus 2 (SARS-CoV-2) [7, 8] and other viruses, such as human papillomavirus (HPV) [9].

Additionally, saliva can also serve as an indicator of oral cavity health, particularly in relation to dental caries, calculus formation, or periodontal diseases caused by microbial infections, such as gingivitis and periodontitis [10, 11]. One of the characteristics of saliva that can serve as a biomarker is its pH value. Salivary pH plays a crucial role in maintaining oral homeostasis and buccal health by promoting enamel mineralization and inhibiting the growth of microorganism under normal conditions. However, factors such as medication, diet, oral hygiene routine and systemic or buccal diseases can alter salivary pH. This acidification or alkalinization of the environment is related to the growth and colonization of buccal surfaces by microorganisms responsible for periodontitis and gingivitis, respectively [10], and the formation of plaque.

In this context, saliva emerges as an ideal candidate for non-invasive monitoring through Point-of-Care (POC) platforms [6, 12]. POC devices are designed for use at home or in a clinical setting and provide real-time results for immediate diagnosis and treatment. Lateral flow devices, which have a paper-based core, and other saliva-based POC systems have been extensively utilized for the diagnosis of SARS-CoV-2 by patients or health professionals during the pandemic. Currently, there is a growing trend in the development and utilization of saliva devices of this nature, incorporating smartphone for diagnosis [13].

In this work, our focus lies in the development and validation of a POC device for in situ determination of oral pH without the need for complex instruments, relying solely on a smartphone as the detection device. This requires the use of a non-toxic indicator immobilized on a membrane that effectively retains the indicator and prevents leaching during sample collection, enabling direct sampling within the buccal cavity.

Water-soluble anthocyanins, which are natural non-toxic dyes and pH-sensitive compounds, serve as an ideal candidate for the proposed device. By combining a membrane that changes colour in response to oral pH with a smartphone capable of capturing and processing an image of such membrane, pH determination becomes feasible. Among the plant-derived natural pigments such as anthocyanins, betalains, carotenoids, and chlorophylls, the former standout due to their wide distribution, extensive consumption by humans, common use as food colourants, and potential as nutraceutical ingredients [14]. Anthocyanins are water-soluble phenolic compounds, specifically glycosylated forms of the aglycones anthocyanidins, belonging to the family of flavonoids, present in all tissues of higher plants. They are responsible for the red, blue, and purple colours observed in fruits, flowers, and vegetative organs [15]. Natural anthocyanins pigments, along with phenolic copigments, form non-covalent complexes that stabilize and modulate the colours observed in flowers, berries, and food products derived from them [16]. Anthocyanins have been used as natural and biodegradable pH indicators for different purposes, including optical pH sensors [17–20], visual pH indicators [21], freshness indicators for food safety [16, 22–24], and films for salivary pH determination [17]. However, the primary application of anthocyanins-incorporated films lies in food packaging to monitor the freshness of food products. Among these films, anthocyanins derived from red cabbage are the most widely employed, as discussed in the article by Abedi-Firoozjah et al. [23]. Nevertheless, various sources have been used for the extraction of anthocyanins, such as blueberry [25], black wolfberry [24], purple cauliflower [26], grape skin [18, 19, 21, 27], black rice bran [28], purple sweet potato [22], purple yam [17], karonda [29], minnieroot [20], and red cabbage [30]. However, the colour exhibited depends on the plant source, composition, and configuration of anthocyanins and their stability are influenced by composition, pH, light, temperature, and structure. Different natural biopolymers have been widely employed to produce films incorporating anthocyanins derived from different biocompatible synthetic polymers, such as poly(vinyl alcohol) or polylactic acid, as well as natural sources. Most of these biopolymers are polysaccharides, such as chitosan, starch, cellulose and, in some cases, polyelectrolyte complexes. Additionally, protein-based biopolymers such as gelatine, zein, or soy protein are utilized. Notably, chitosan stands out as the most commonly used natural biopolymer as a matrix for natural dyes [18].

**Fig. 1:**
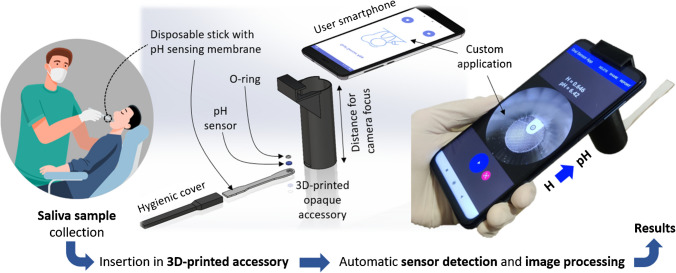
System overview showing the developed sensing device to measure saliva pH directly from the sample collected in the mouth by means of a smartphone-based platform, which consists of a 3D-printed accessory and a custom-developed smartphone application that implements automatic image processing

In this work, we propose a smartphone-based platform [31] consisting of a custom application created in combination with a 3D-printed accessory, designed to hold a disposable stick containing a chitosan-based pH sensing membrane with anthocyanins extract, as presented in Fig. 1. The developed system enables the user to capture an image of the pH sensing membrane, implementing image processing algorithms to automatically identify the pH sensing membrane, compute its internal colour coordinates, and provide the resulting pH value based on a previously stored calibration curve. Additionally, the application allows data storage and results sharing. The designed 3D-printed accessory not only positions the stick, and consequently, the pH sensing membrane, in alignment with the smartphone camera, but it also isolates the region of interest from the external ambient light. By utilizing the camera flash to capture the images, the light conditions under which the pH sensor is always photographed are constant, ensuring consistent results regardless of external lighting conditions.

## Materials and methods

### pH sensor preparation

Two different sensing cocktails were prepared, using chitosan and pullulan as membrane polymers. The chitosan-based cocktail was prepared at a final concentration of 0.5% (w/v) in a 2 M acetic acid solution by mixing in a vortex until achieving homogeneity. On the other hand, the pullulan-based cocktail was prepared by dissolving it in water with the help of an ultrasonic bath to achieve a final concentration of 40 mg/mL. Then, anthocyanins extract was added to raise the final concentration of both cocktails to 20 mg/mL. For the fabrication of the sensing membranes, 5 µL of the sensing cocktail was spotted onto round piece of Whatman 1 paper with a diameter of 2.5 mm, obtained from a sheet by using a punch. The sensing membranes were then dried on a stove at 45º C for 30 min and subsequently stored overnight in a refrigerator at 4º C. Finally, the device used to measure the pH was assembled by placing the sensing membrane in the disposable stick with the aid of the O-ring to adjust the holder’s location (see Fig. 1).

### Analytical protocol

The device is used by directly moistening the membrane in the oral cavity, following the Standard Operational Procedure (SOP) for sample collection (refer to Section S14 of the Supplementary Information). After sample collection, the analytical device is inserted into the sampling hole of the 3D-printed black accessory, where the smartphone is attached to secure it in position and maintain darkness. Each image was captured using the custom-developed smartphone app after 60 s of sample collection with the flash mode enabled. As detailed in Section “Image acquisition and processing”, the app automatically processes the image to identify the circular-shaped pH sensing membrane and transforms its internal RGB colour coordinates into HSV to derive the Hue (H) value of the ROI using an automated algorithm. Then, the app automatically correlates the H value with the pH of the sample. More details regarding the selection of the H value as the analytical parameter are given in Section “Analytical parameter selection”.

### Analysis of real samples

The study was conducted in accordance with the guidelines of the Declaration of Helsinki and received approval from the Ethics Committee in Human Research (CEIH) of the University of Granada (2840/CEIH/2022). Informed consent was obtained from all participants involved in the study. Real saliva samples were collected from subjects without known diseases, adhering to the SOP described in Section S14 of the Supplementary Information. To obtain reference measurements, a previously calibrated pH meter was used by introducing the electrode directly into the Eppendorf and taking three replicated measurements.

## Results and discussion

### Chromogenic reagent selection

The objective of this study is to design and characterize a sensing device capable of directly measure saliva pH in the mouth using a simple method with the assistance of a smartphone-based platform. The device is based on acidochromism originating from a sensor, which employs an immobilized acidochromic indicator dye as recognition element. Anthocyanins were chosen as the natural indicator dye due to their approved for use as food colourants in the European Union, Australia, and New Zealand, with the colourant code E163 [32]. In general, anthocyanins appear red at acidic pH lower than 3 due to the flavylium cation; violet at pH levels between 6 and 7 due to the quinoid anhydrobase; blue at pH levels between 7 and 8 blue due to the anionic quinone; and blue-green at pH levels higher than 11 due to the dianionic form [15].

Red cabbage was selected as the source of anthocyanins due to its higher concentration compared to other plants, although this concentration varies depending on the variety, agricultural practices, and maturation time [33]. Red cabbage contains more than 30 anthocyanins, most of which are derivatives of cyanidin-3-diglucoside-5-glucoside in non-acylated, mono-acylated and diacylated forms with ferulic, sinapic, p-coumaric, and caffeic acids [34]. The high presence of acylated anthocyanins enhances the thermal and photo stability of the dye [35]. In this case, the extraction of anthocyanins from red cabbage was carried out using purified water, without the usual assistance of organic solvents.

### Analytical parameter selection

The first conducted test involved the preparation of pullulan and chitosan-based sensing membranes containing anthocyanins, and the characterization of their colour change within the pH range of 4–9 in terms of colour coordinates using the RGB and HSV colour spaces. For this purpose, the procedure described in Section S4 of the Supplementary Information was followed, and the obtained results are presented in Fig. S1.

Among all the colour coordinates investigated, only the red (R) value from the RGB space and the Hue (H) value from HSV space exhibited significant changes with pH, showing a sigmoidal dependence. No variation was observed in the case of green (G) and blue (B) coordinates when the pH changed. The difference in the H value between the tested pH extremes was found to be 9.3% for the range of pH 4 and 9, and 6.6% for the range of pH 6 to 8, with coefficient of variation (CV) values of 0.3% and 0.3%, respectively. In the RGB space, the R coordinate yielded a variation of 9.0% with a CV of 1.4% for the pH range of 4 to 9, and 6.1% with a CV of 1.4% for the pH range of 6 to 8 in the pullulan-based cocktail.

For the chitosan-based cocktail, the difference in the H value between the pH extremes tested was 10.5% for the pH range of 4 and 9, and 6.9% for the pH range of 6 to 8, with CV values of 0.8% and 0.7%respectively. In the RGB space, the R coordinate exhibited a variation of 10.5% with a CV of 2.5% for the pH range of 4 to 9, and 6.7% with a CV of 2.1% for the pH range of 6 to 8.

According to the obtained results, The H coordinate was finally selected as the analytical parameter due to its high reproducibility, lower CV, and higher sensitivity to pH compared to the R coordinate. This can be observed in Fig. S1 for both the pullulan-based and chitosan-based cocktails.

### Polymer retention capacity and concentration optimization

When pH indicators are used on a cellulose support, most of them need to be included in a polymeric membrane to prevent changes in the pK_a_ and ensure a consistent response range. However, for our device, we required a method that could be used by introducing the device into the mouth. To achieve this, the chromoreagent must be immobilized to prevent leaching from the device into the sample, which can cause irreproducibility. For this reason, two biocompatible polysaccharide polymers were selected in this study, pullulan and chitosan. These polymers were evaluated as retained polymers to enhance the homogeneity of the membrane and avoid undesirable effects such as the coffee ring effect or a decrease in the reproducibility of the entire method due to anthocyanins migration once the sample is spotted.

**Fig. 2:**
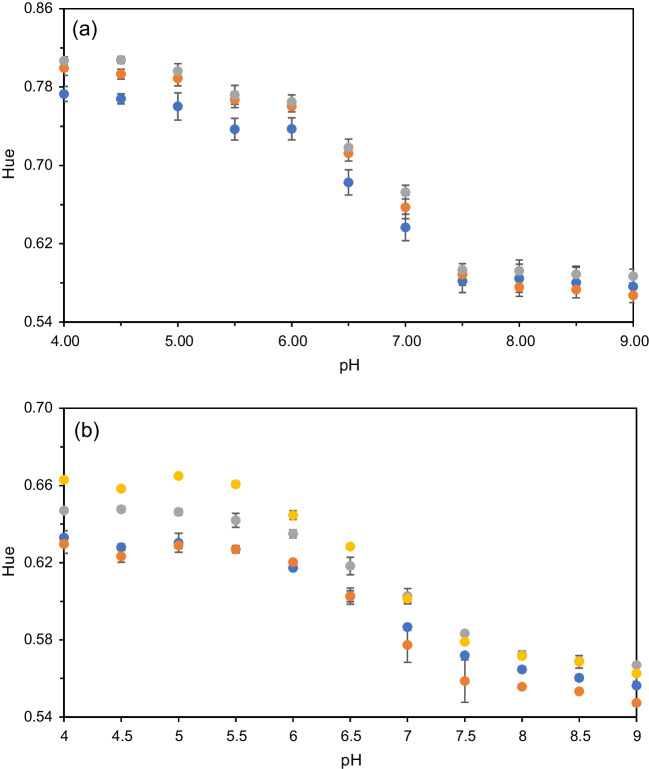
**a** H coordinate variation when 0.25 (blue dots), 0.50 (orange dots) and 1.00% (w/v) (grey dots) of chitosan and 15 mg/mL of anthocyanins extract are tested. **b** H coordinate variation when 10 (blue dots), 25 (orange dots), 40 (grey dots) and 50 mg/mL (yellow dots) of pullulan and 15 mg/mL of anthocyanins extract are tested

The polymer concentration in the cocktail was also optimized because the membrane properties are dependent on the polymer concentration in terms of wetting angle, hydrophilicity, reaction time, and the distribution of anthocyanins across the sensing membrane. Additionally, the optimal polymer concentration is critical for retaining the anthocyanins while maintaining the wetting properties of the membrane. Therefore, membranes with varying concentrations of pullulan and chitosan were prepared and tested as described in Section S5 of the Supplementary Information. For chitosan (see Fig. 2(a)), concentrations of 0.25%, 0.50%, and 1.00% (w/v) were tested, resulting in H coordinate variations (ΔH) from pH 6.0 to 8.0 of 0.150, 0.180, and 0.170, respectively. For pullulan (see Fig. 2(b)), the variations of H between pH 6.0 and 8.0 for concentrations of 10 mg/mL, 25 mg/mL, 40 mg/mL, and 50 mg/mL were 0.065, 0.073, 0.074, and 0.093 respectively. The optimal concentrations for the chitosan- and pullulan-based cocktails were found to be 0.5% (w/v) and 40 mg/mL, respectively, achieving the best balance between H change and pH indicator retention in the assay with loaded strips (Fig. S2) described in Section S6 of the Supplementary Information. In fact, these concentrations were proven to be the most effective in both retaining anthocyanins capacity and maintaining wet and flow characteristics in the retention assay. The chitosan-based cocktail at a concentration of 0.5% (w/v) (Fig. S3 and Table S1) was able to retain 84.2% of its initial anthocyanins, and the pullulan-based cocktail at a concentration of 40 mg/mL (Fig. S4 and Table S2) retained 86.3% in this assay when a flow of 40 µL of buffer solution crossed the sensing zone. Higher concentrations of the polymers did not significantly improve retention but had a negative impact on the microfluidics characteristics.

### Anthocyanins concentration optimization

The next parameter to be optimized was the anthocyanins extract concentration, as it plays a crucial role in obtaining the optimal signal variation and, consequently, analytical parameters for the device. The optimization process is described in Section S7 of the Supplementary Information, where it was determined that a concentration of 20 mg/mL of anthocyanins extract yielded the best results, considering both the signal variation and the CV obtained from three replicates (Fig. S5). At this concentration, the device exhibited the best performance, with a signal variation of 0.104 in the H value between pH 4 and 9, and a CV of 0.2%. Using a concentration of 25 mg/mL resulted in a variation of 0.099 and a CV of 0.2%, while at 15 mg/mL, the variation in H was 0.080 with a CV of 0.3%. In the case of 10 mg/mL, the signal variation was considerably lower and could adversely affect the analytical parameters of the device.

Once the cocktail components were optimized and tested, and their optimal performance concentrations were determined, the total amount of cocktail required to load the paper-based sensor was also optimized (Section S8 of the Supplementary Information). A total volume of 5 µL (Table S3) achieved the highest variation within the pH range of interest.

**Fig. 3:**
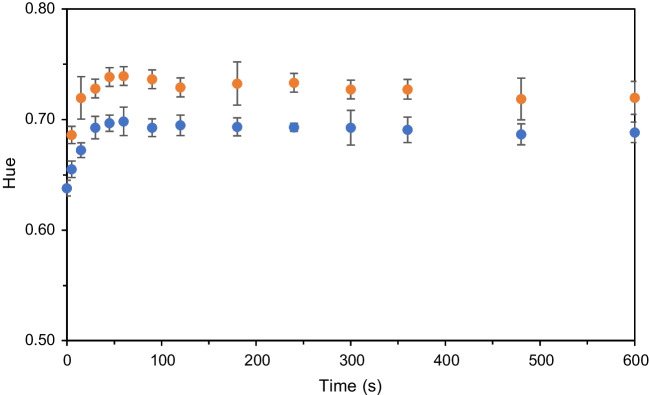
Hue values of the pullulan-based (blue dots) and chitosan-based (orange dots) cocktail at a concentration of 40 mg/mL and 0.5% (w/v) with 20 mg/mL of anthocyanins extract at different time from 0 to 600 s when the device was tested with pH 4

Finally, a kinetic study was performed as described in Section S9 to determine the time required to obtain a steady signal at pH 4 (See Fig. S6 for pullulan assay). The study revealed that both polymers required 60 s to obtain a steady signal that remained unchanged until 150 s (see Fig. 3). After that time, the signal began to change due to the drying process, which altered the colour of the device.

### Sensing membranes analytical characterization

Once the sensing membranes were optimized, they were calibrated and analytically characterized (Section S10) using the digital camera in the lightbox, as described in Section S3. For this purpose, 19 different standard solutions (with three replicates each) were employed to obtain the calibration function by adding 2 µL of standard onto the sensing membrane. The sensing membranes exhibited a sigmoidal behaviour (refer to Fig. S7) that was fitted to a Boltzmann equation (Equation S1). The analytical parameters for both types of sensing membranes, as indicated in Table S4, demonstrate the ability to determine pH within the respective ranges of 5.5 to 7.8 for pullulan-based membranes, and 5.4 to 7.8 for chitosan-based membranes. Finally, precision was assessed in terms of CV, yielding a value of 0.2% for pullulan-based membranes and 0.6% for chitosan-based membranes.

### Smartphone-based platform performance

Once the pH sensing membranes were optimized and characterized, the next step was to incorporate them into the designed disposable stick to securely hold them, thus allowing easy collection of saliva directly from the oral cavity. This device, in combination with the 3D-printed accessory (Fig. S9) and the custom-developed smartphone application, enables the automatic detection of the pH sensing membrane by means of image processing algorithms, the calculation of its colour coordinate (i.e., the H value), and the final correlation it to the buccal pH value.

#### Image acquisition and processing

**Fig. 4:**
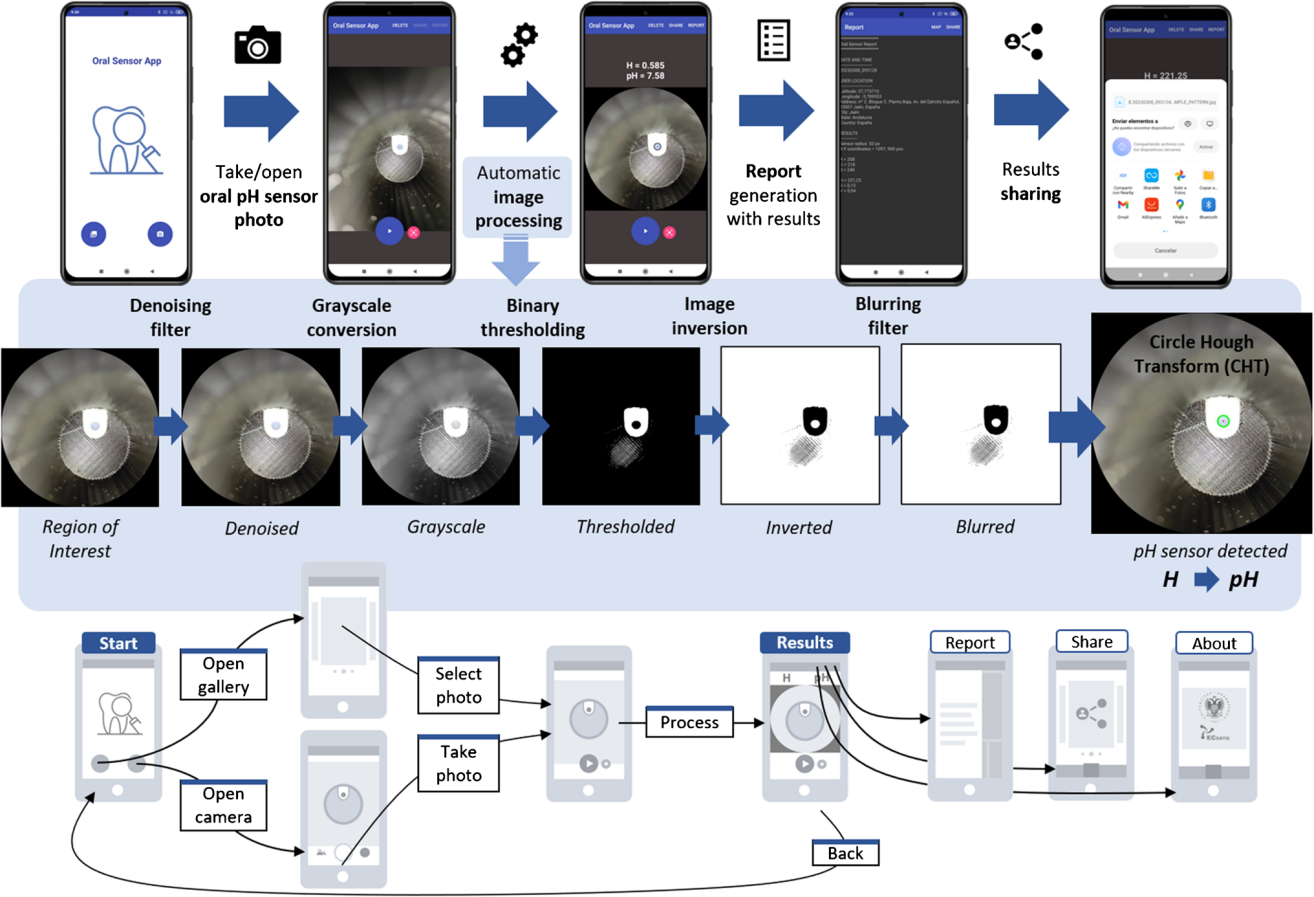
Flow diagram describing the image processing sequence conducted by the custom smartphone application, including screen captures of the key steps and the application user flow for the automatic acquisition and processing of the pH sensing device

The entire process is illustrated in the application user flow of Fig. 4. Initially, the welcome screen displays a menu with two options: *(1)* Select a photograph from the gallery, or *(2)* Take a photograph. If the latter is selected, the application automatically starts the rear camera of the device so that the user can take a photograph of the pH sensing membrane. To take the photograph, the app activates the device's rear camera, allowing the user to photograph the pH sensing membrane with specific setup parameters, including a resolution of 4000 × 3000 pixels, f/1.83 aperture value, 1/40 s exposure time, ISO 800, and auto-focus. After selecting or taking the photograph, the user can initiate automatic image processing by clicking on the *Process* button, which is graphically summarised in Fig. 4. This processing involves using the Circle Hough Transform (CHT) to detect the circular colourimetric sensor. CHT is a feature extraction technique utilized in digital image processing to identify circular objects in imperfect digital images [36]. Our group has already successfully employed this technique in previous studies [37]. Before the CHT detects the circle, a pre-processing stage is necessary to prepare the image. Firstly, the image is denoised, and then converted to grayscale since subsequent functions require a grayscale image as input. Following this, we experimentally verified that binary thresholding, followed by inversion of the image, enhanced the circle's detection algorithm's reliability and accuracy. After performing these operations, a low-pass blur filter smooths the edges and removes the image's noise. Ultimately, OpenCV's CHT function was used to automatically detect the pH sensor. Upon detection, a separate mask is applied, which eliminates everything from the image except the detected circle. In creating each mask, the circle's radius is decreased by 20% to ensure that the sensor's colour is entirely detected inside the circle if the edges are blurry. After the image processing, the application provides information related to the detected pH sensor, including its average RGB and HSV coordinates. Subsequently, the pH value is computed based on the corresponding calibration curve (refer to Section System calibration, stability and performance). When the entire processing pipeline is complete, the app generates a report in plain text, which is stored in the phone's memory, containing all the collected information about the detected pH sensor. The results (both image and report) can be shared via email and/or different messaging services. More details regarding the image processing pipeline are given in Section S12 of Supplementary Information. A demonstration video is included as Supplementary Video V1.

#### System calibration, stability and performance

**Fig. 5:**
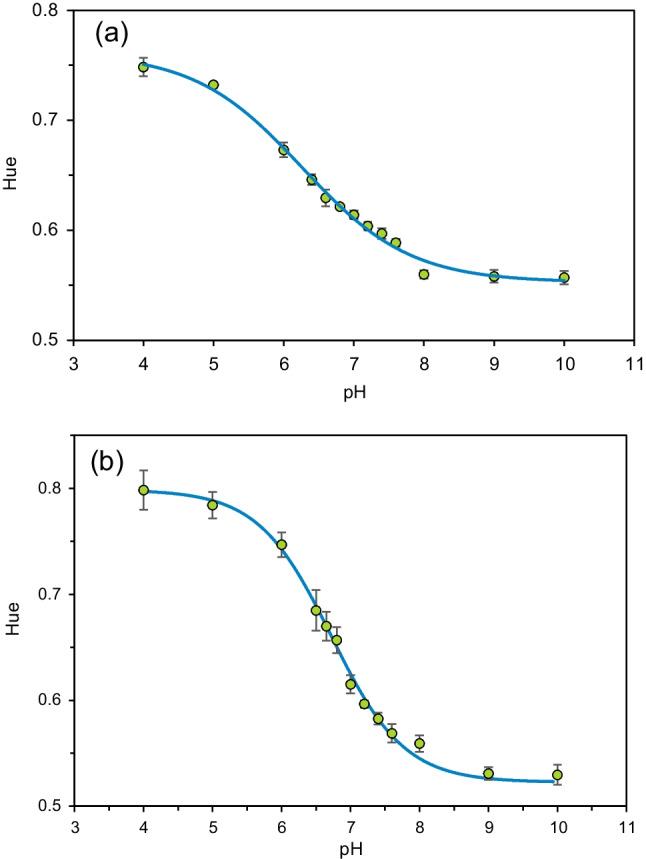
Calibration curve of the pullulan-based (**a**) and chitosan-based (**b**) cocktail obtained with the smartphone-based platform

The pH sensing membranes were calibrated using the described smartphone-based platform. For this purpose, we employed 13 pH standards, each with three replicates, as detailed in Section S11. The resulting calibration function, along with the obtained dataset, is presented in Fig. 5, while the analytical parameters are summarized in Table S5. By utilizing the 3D-printed add-on accessory with the automated system for the ROI detection and the H parameter calculation, we successfully minimized the analysis time required for each experiment. As a result, the CV for pullulan membranes was 0.77%, and 1.58% for chitosan membranes. The working range was found to be from 4.9 to 8.0 for the pullulan-based membranes and from 5.4 to 8.1 for the chitosan-based ones. To validate the technical reproducibility of the developed system, we captured three different photographs of the sensing membranes for each pH value and processed them independently. For both polymers-based cocktails, the obtained technical reproducibility exceeded 99%, as explained in Section S11. It is worth mentioning that if a different smartphone model was used instead of the one employed in our study, it would be necessary to repeat the calibration of the sensing membranes due to the variations in the camera characteristics.

An additional assay was conducted to investigate the device’s stability over time. For this purpose, 60 analytical devices were placed in 20 sealed aluminium/Mylar bags filled with N_2_ and stored in a refrigerator at 4ºC. The stability test spanned 84 days, during which the devices were tested 20 on different days by adding a pH 7 standard solution and measuring the resulting signal. As shown in Fig. S8, the devices remained stable until day 50, after which the signal may exhibit lower than expected values, potentially compromising the results.

The analytical device was validated using the smartphone equipped with the support and the sticks and compared to the calibrated pH-meter results. The SOP described in Section S14 of the Supplementary Information was followed for sample collection and individual exclusion. The average error between the reference values obtained was 2.4% for the chitosan-based cocktail (Table S7) and 11.4% for the pullulan-based cocktail (Table S8), exhibiting a positive systematic error. This systematic error, which leads to salivary pH values higher than expected, may be attributed to the partial digestion of pullulan by human salivary enzymes, such as, α-amylase [38]. In conclusion, the anthocyanins-containing chitosan membrane spotted on paper was selected as the optimal method for measuring buccal pH using saliva as a sample, in combination with the smartphone equipped with the designed add-on and running the custom image-processing application.

## Conclusions

A practical, green, and user-friendly device is proposed for the direct determination of buccal pH, utilizing a smartphone and a 3D-printed add-on. The device incorporates an edible colourimetric indicator, namely anthocyanin, extracted from red cabbage (*Brassica oleracea*), which is immobilized on a polysaccharide membrane within a single-use stick. This design allows for convenient and direct saliva sampling within the oral cavity. Subsequently, the disposable stick is inserted into the add-on, where the smartphone camera captures an image and performs automatic sensor detection and processing to provide the salivary pH results in just 1 min.

Chitosan and pullulan were employed for anthocyanin immobilization with favourable results after the optimization of different variables against standard buffers. However, membranes prepared with pullulan exhibited a systematic error when applied to real saliva samples. In contrast, when chitosan was used as the polymer, this systematic error was eliminated. The chitosan-based membrane was validated against a group of volunteers, with a calibrated pH-meter serving as the reference, resulting in an error of approximately 2%.

The designed device for direct analysis of biomarkers within the oral cavity holds the potential to pave the way for new chemistries in POC format, as well as facilitate practical zonal sampling of the mouth.

### Supplementary Information

Below is the link to the electronic supplementary material.

Supplementary file1 (PDF 596 KB)

Supplementary file2 (MP4 9893 KB)

## Data Availability

The data that supports the findings of this study are available in the supplementary material of this article. Data available on request.
