# Supplementary material for: POC device for rapid oral pH determination based on a smartphone platform
Source: Mikrochim Acta. 2024 Feb 14;191(3):134. doi: 10.1007/s00604-024-06227-1 (PMC10867041; doi:10.1007/s00604-024-06227-1)
Supplement: Supplementary file 1 — Supplementary file1 (PDF 596 KB) [file 604_2024_6227_MOESM1_ESM.pdf]

Supplementary Information

POC device for rapid oral pH determination based on a smartphone platform.

Manuel J. Arroyo[a], Pablo Escobedo[b,d*], Isidoro Ruiz-García[b], Alberto J. Palma[b,d] Francisco Santoyo[c,d], Mariano Ortega Muñoz[c,d], Luis Fermín Capitán-Vallvey[a,d], Miguel M. Erenas[a,d*]

[a] ECsens, Department of Analytical Chemistry, Campus Fuentenueva, University of Granada, Granada, Spain.

[b] ECsens, CITIC-UGR, iMUDS, Department of Electronics and Computer Technology, University of Granada, Granada, Spain

[c] Department of Organic Chemistry, Campus Fuentenueva, University of Granada, Granada, Spain.

[d] Unit of Excellence in Chemistry applied to Biomedicine and the Environment of the University of Granada.

*Corresponding authors

Email address: erenas@ugr.es (Miguel M. Erenas) and pabloescobedo@ugr.es (Pablo Escobedo)

Table of contents

S1. Reagents and materials 2
S2. Red Cabbage extract 3
S3. Instruments and software 3
S4. Analytical parameter determination 4
S5. Polymers concentration optimization. 6
S6. Polymers retention power and concentration optimization 6
S7. Anthocyanins concentration optimization 10
S8. Sensor cocktail volume optimization 11
S9. Reaction Kinetics 11
S10. Calibration with camera setup 12
S11. Calibration with smartphone and custom app 14
S12. Automatic image processing algorithm 15
S13. Stability performance 18
S14. SOP for sample collection and individual exclusion 18
S15. Validation of polymers-based cocktail with smartphone app 19
S16. 3D-printed accessories 20

S1. Reagents and materials

All the reagents used for cocktail and solutions preparation were of analytical grade and were purchased from Sigma-Aldrich (Madrid, Spain). As explained below, anthocyanins extract was obtained through extraction methods from red cabbage (Brassica oleracea var. capitata f. rubra) purchased at a local grocery store (Hipercor, Granada, Spain). Other chemicals used included pullulan (maltotriose trimer) (CAS No. 9057-02-7; E1204), chitosan (poly-(D)glucosamine) (CAS No. 9012-76-4), and acetic acid. Whatman® nº 1 (Whatman, UK) was used as support for the pH sensing membrane. All the aqueous solutions were prepared using purified water with a resistance of 18.2 MΩ·cm, obtained from a Milli-RO 12 plus Milli-Q station (Millipore, Bedford, MA, USA). Different pH standard solutions ranging from 4 to 10, used for calibration purposes of the device, were prepared using phosphate buffer (NaH2PO4/Na2HPO4) in purified water.

S2. Red Cabbage extract

Red cabbage leaves were cut into small pieces. Extraction was conducted by combining 500 g of these leaves with 1000 mL of water. The beaker was gradually heated to 70 ºC and maintained at this temperature for 15 minutes. After the coloured liquid had cooled, it was strained through filter paper. The solution was then evaporated using a vacuum evaporator system at 40º C, reducing the volume to one-fourth of the initial amount. The resulting concentrated solution was freeze-dried to obtain a purple solid, which was stored in a freezer at -20ºC.

S3. Instruments and software

For calibration purposes, pH measurements were conducted using a XS PH 8 + DHS STIRRER pH bench meter (XS Instrument, Italy) with a 238100 MiniTrode (Hamilton, USA) electrode. To maintain a nitrogen atmosphere during the stability assay, the sensor devices were stored in Albal freezer polyethylene (PE) bags with three layers (Cofresco Ibérica, S.A.U., Spain).

The disposable stick to hold the sensing membrane as well as its accessories (i.e., the hygienic cover and the O-ring) were 3D-printed with a Creality CR-X 3D printer (Creality, Shenzhen,China) using polylactic acid (PLA) white filament.

During the study, the pH sensor membrane was digitalized both using a digital camera and the smartphone-based platform. To maintain control over image acquisition features and eliminate any external lighting conditions when utilizing the digital camera, it was located in a fixed position inside a cubic lightbox equipped with two panels, each containing 50 LEDs (550 lumens; colour temperature: 5600K). For still images, a Sony DSC-HX300 camera (Sony, Tokyo, Japan) was employed, with a resolution of 3648 x 2736 pixels, f/4 aperture value, 1/40 s exposure time, ISO 80, and 5600 K white balance. The captured images were saved in Joint Photographic Experts Group (JPEG) format (). For video recordings, the camera was configured to a resolution of 1440 x 1080 pixels, 25 frames per second, and 5000 K white balance, and the file was saved in MTS (AVCHD) format. Avidemux 2.6 (free software developed by "Mean", "Gruntster" and "Fahr") was employed to extract individual frames from the recorded videos. Both the

images obtained from video captures and those directly from the camera were analysed using ImageJ software (National Institutes of Health). To maintain consistency across all measurements, the circular region of interest (ROI) of the sensing membranes was manually selected, and ROI templates were saved. The templates contained 9768 pixels for images and 1988 pixels for video captures, providing the RGB and HSV chromatic coordinates of each pixel, along with the average ROI values. To perform statistical and mathematical treatment, OriginPro v.8 software (OriginLab Corporation, USA) and Microsoft Excel (Microsoft, USA) spreadsheets were utilized.

In the case of the smartphone-based platform, the digitalization of the membranes within the µPAD devices was performed using the 3D-printed accessory in combination with a Xiaomi Redmi Note 9 Pro (M2003J6B2G, Xiaomi, China) smartphone running the custom-developed Android application The smartphone application was programmed using the Integrated Development Environment (IDE) Android Studio Dolphin 2021.3.1. The app was designed and tested for compatibility with Android 12, which corresponds to API level 31. Nevertheless, it can also run on earlier Android versions, with the minimum requirement being Android 6.0 (API level 23). For image acquisition and processing, the open-source OpenCV 3.1.0 library was employed

S4. Analytical parameter determination

To determine the analytical parameter, a set of pH standard solutions (4, 4.5, 5, 5.5, 6, 6.5, 7, 7.5, 8, 8.5, and 9) consisting of 0.1 M phosphate buffer were spotted using 5µL of the volume of each solution in three membranes as replicates. Both chitosan and pullulan-based cocktails were evaluated. One set of sensing membranes was loaded with 2 µL of a pullulan-based cocktail containing 40 mg/mL of pullulan and 15 mg/mL of anthocyanins extract, while the second batch was coated with 0.5% (w/v) of chitosan and 15mg/mL of anthocyanins extract (Fig. S1).

Sixty seconds after adding 2 µL of the sample, the sensing membrane was photographed using a digital camera placed in a white light box with the setup described in the ‘Image acquisition and processing’ section of the main manuscript. The images were analyzed using Image J software to obtain the region of interest (ROI), which encompassed approximately 9780 pixels. The RGB colour coordinates were also extracted. Furthermore, the Hue coordinate (H) was calculated by converting the colour space of the

images from RGB to HSV. For that purpose, the buffer solutions were spotted, and captures were taken 60 seconds after that. The same circular ROI of 9768 pixels was used for analysis in both RBG and HSV colour spaces.

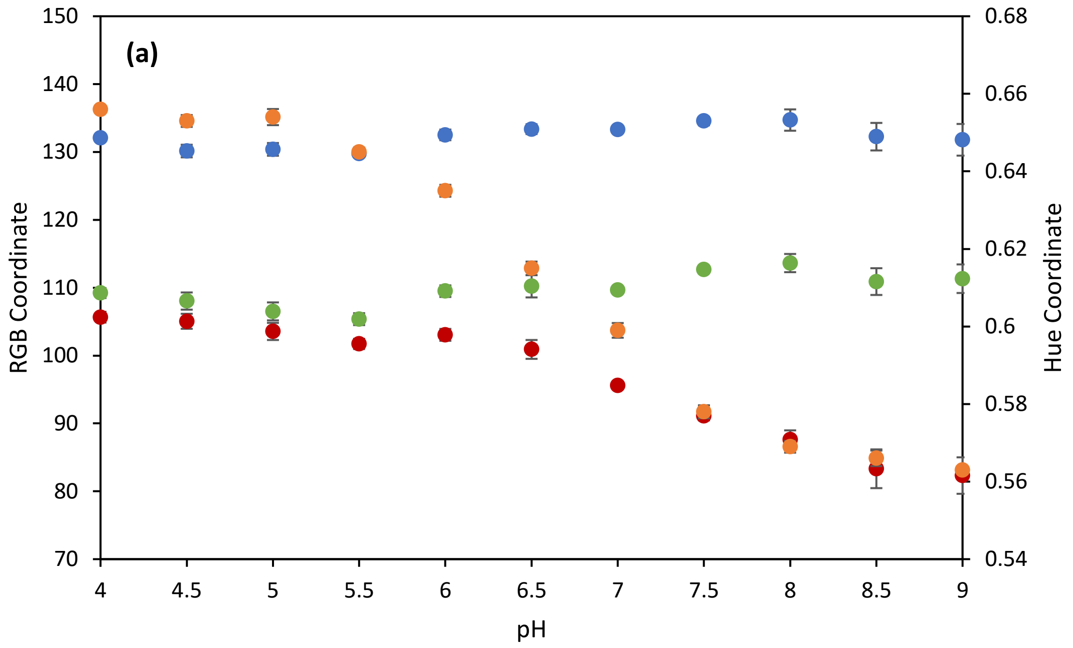

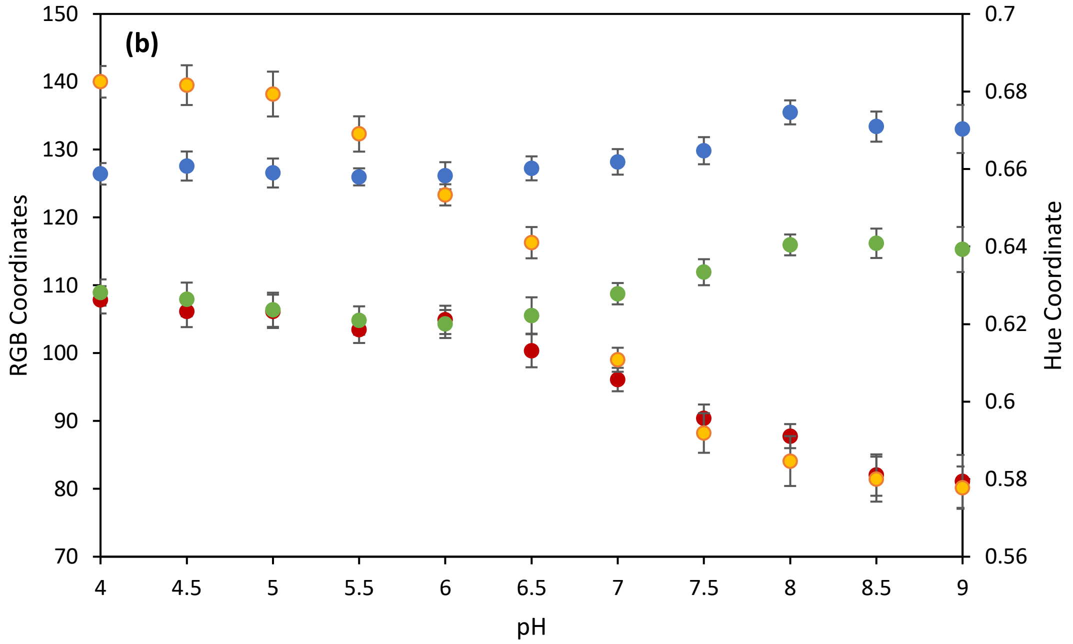

Fig. S1. a) RGB (red, green, and blue symbols) and H (orange symbols) coordinates values of the pullulan-based cocktail at 40 mg/mL and 15 mg/mL of anthocyanins extract at different pH buffer solutions. **b)** RGB (red, green, and blue symbols) and H (orange symbols) coordinates values of the chitosan-based cocktail at 0.5% (w/v) and 15 mg/mL of anthocyanins extract at different pH buffer solutions.

S5. Polymers concentration optimization.

The optimization of pullulan and chitosan concentrations in the cocktail was essential due to the potential interference of the polymeric matrix. The presence of these polymers could impact various properties of the paper, such as wetting angle, hydrophilicity, reaction completion time, and even the distribution of anthocyanins across the sensor zone, thereby directly influencing the obtained H values. In addition, unsuitable polymer amounts or concentrations might result in insufficient anthocyanins retention or hinder the wetting of the sensor, respectively.

To determine the optimal concentrations of chitosan and pullulan that would yield the best wetting properties and the highest H variation, 5 µL of 0.25%, 0.5%, and 1% (w/v) solutions of chitosan and 10, 20, 40, and 50 mg/mL solutions of pullulan were tested with pH values ranging from 4 to 9, each containing 15mg/mL of anthocyanins extract concentration (see Fig. 2). For each concentration, three replicates were performed, and the measurements were taken after 60 seconds of adding 2 µL of buffer. The pH scale was incremented by 0.5 units for each measurement.

S6. Polymers retention power and concentration optimization

Three paper strips, each with a width of 2.5 mm and sufficient length, were loaded with 10 µL of chitosan and pullulan concentration containing 20 mg/mL of anthocyanins extract at the beginning of the strip to create a sensor zone. The concentrations tested were 0.25%, 0.5%, and 1% (w/v) for the chitosan based-cocktail, and 10, 25, 40, and 50 mg/mL for the pullulan based-cocktail. After being left to dry overnight, 40 µL of pH 4 0.1 M phosphate buffer was spotted at the beginning of the cocktail zone. Through capillary action, the buffer solution permeated towards the end of the strip passing through the cocktail zone. To measure the quantity of cocktail with the anthocyanins dragged by the flow of the solution, the saturation (S) plot profiles were obtained for a rectangular region of interest corresponding to 20590 pixels, which matched the strip size (Fig. S2), using ImageJ software. These images were captured using Avidemux from the recorded videos and saved in jpeg format. The plot profiles were analyzed at two time points: when the pH 4 solution was spotted and the entire sensor zone had undergone a colour change, and 60 seconds later (Fig. S3 and S4). To evaluate the anthocyanins dragged by de pH 4 solution, the difference in the area under the curves (AUC) of the S

plot profiles for the sensor zone was calculated between time 0 and 60 seconds (Tables S1 and S2).

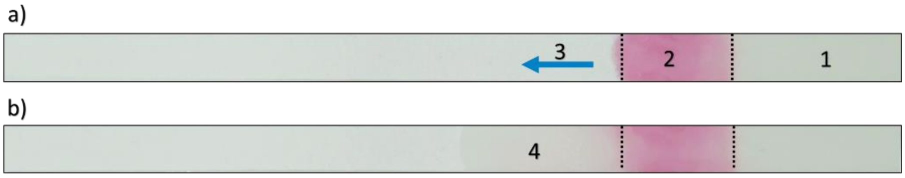

Fig. S2. Example of paper strip loaded with a polymer-based cocktail containing 40 mg/mL of anthocyanins extract in the right zone for retention analysis. Initially, the sample was deposited in Zone 1, and flowed towards the sensing zone (Zone 2) for saturation measurement at time 0, when the flow achieved the final area of Zone 2. The flow then proceeded to the left (Zone 3). At time 60 seconds (b), the dragged anthocyanins had traverses Zone 4.

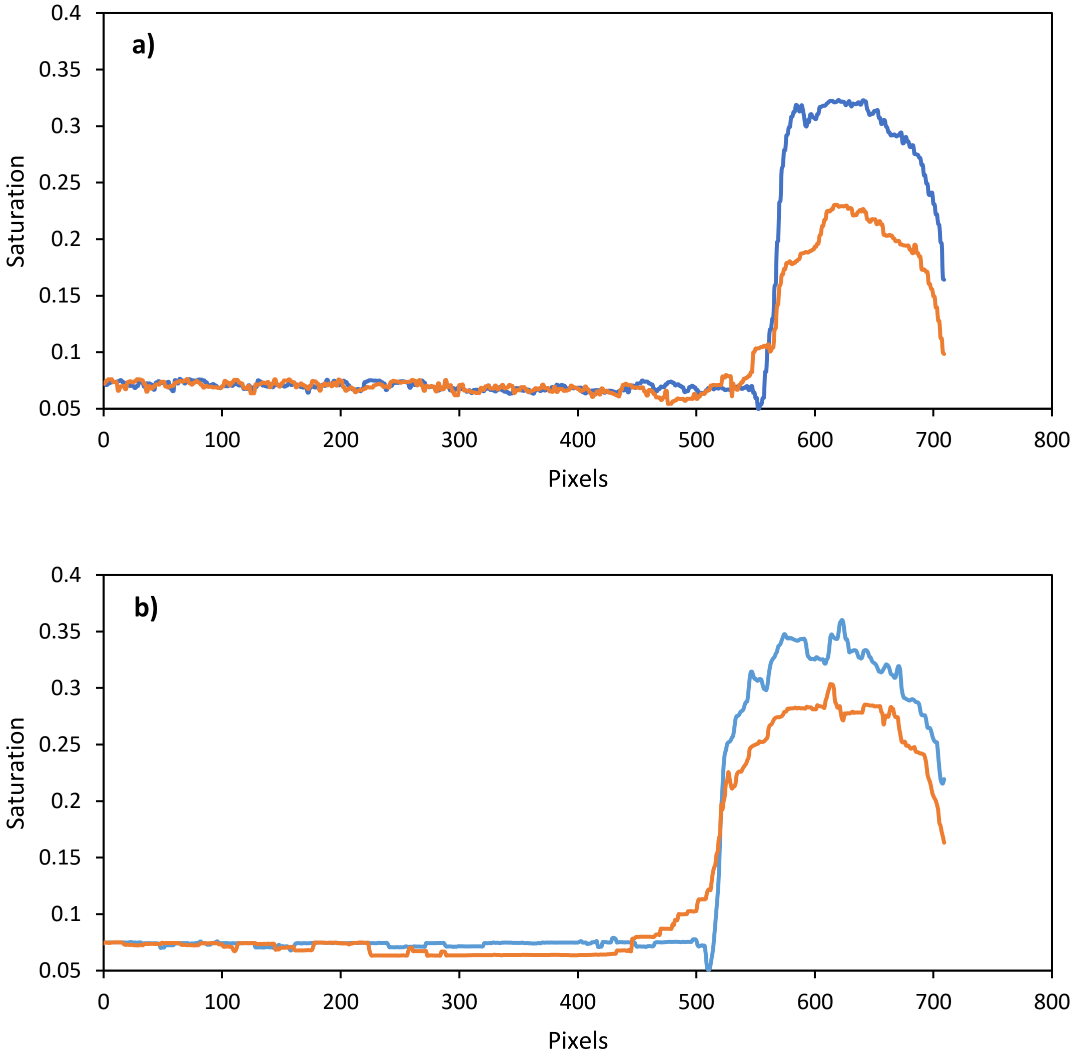

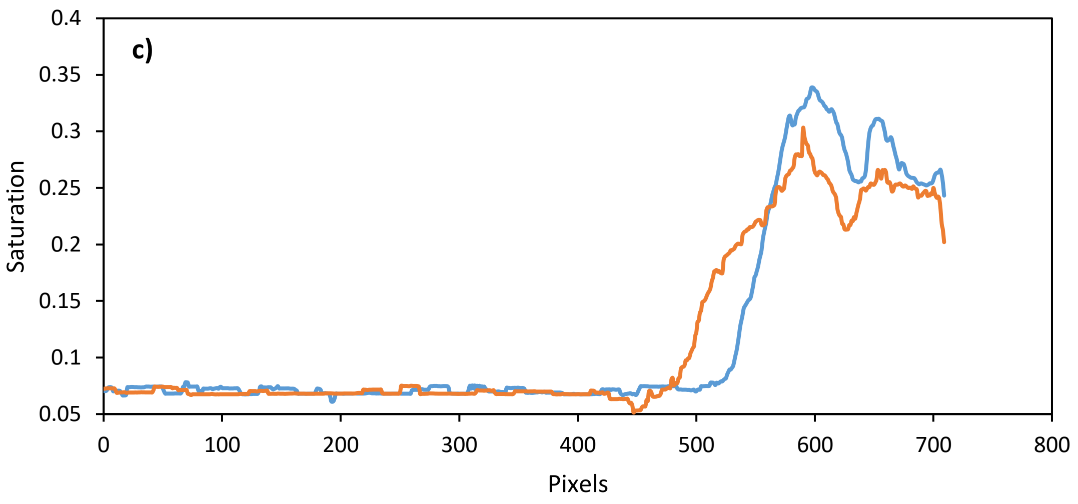

Fig. S3. Plot saturation profile through the paper strip (distance in pixels) before (blue) and after 60 seconds (orange) dropping buffer solution with **A**: 0.25, **B**: 0.5 and **C**: 1% (w/v) of chitosan-based cocktails.

Table S1. Anthocyanins dragged from sensor zone with chitosan as retention polymer in saturation variation terms.

Chitosan Concentration % (w/v)	% Anthocyanins Dragged from sensor zone (difference between time 60 and 0 of Saturation) / Anthocyanins Retained
0.25	32.3 % / 67.8%
0.50	15.7 % / 84.2%
1.00	12.2% / 87.7%

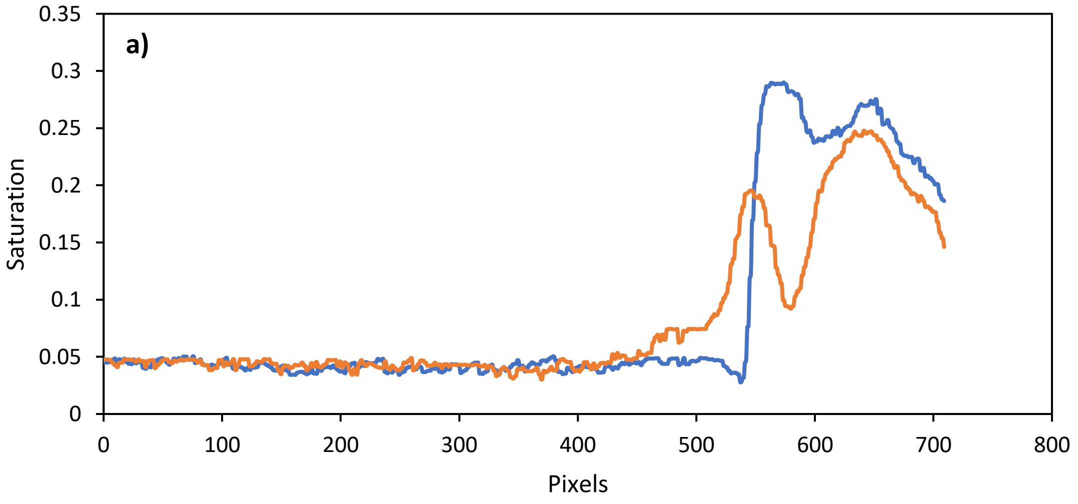

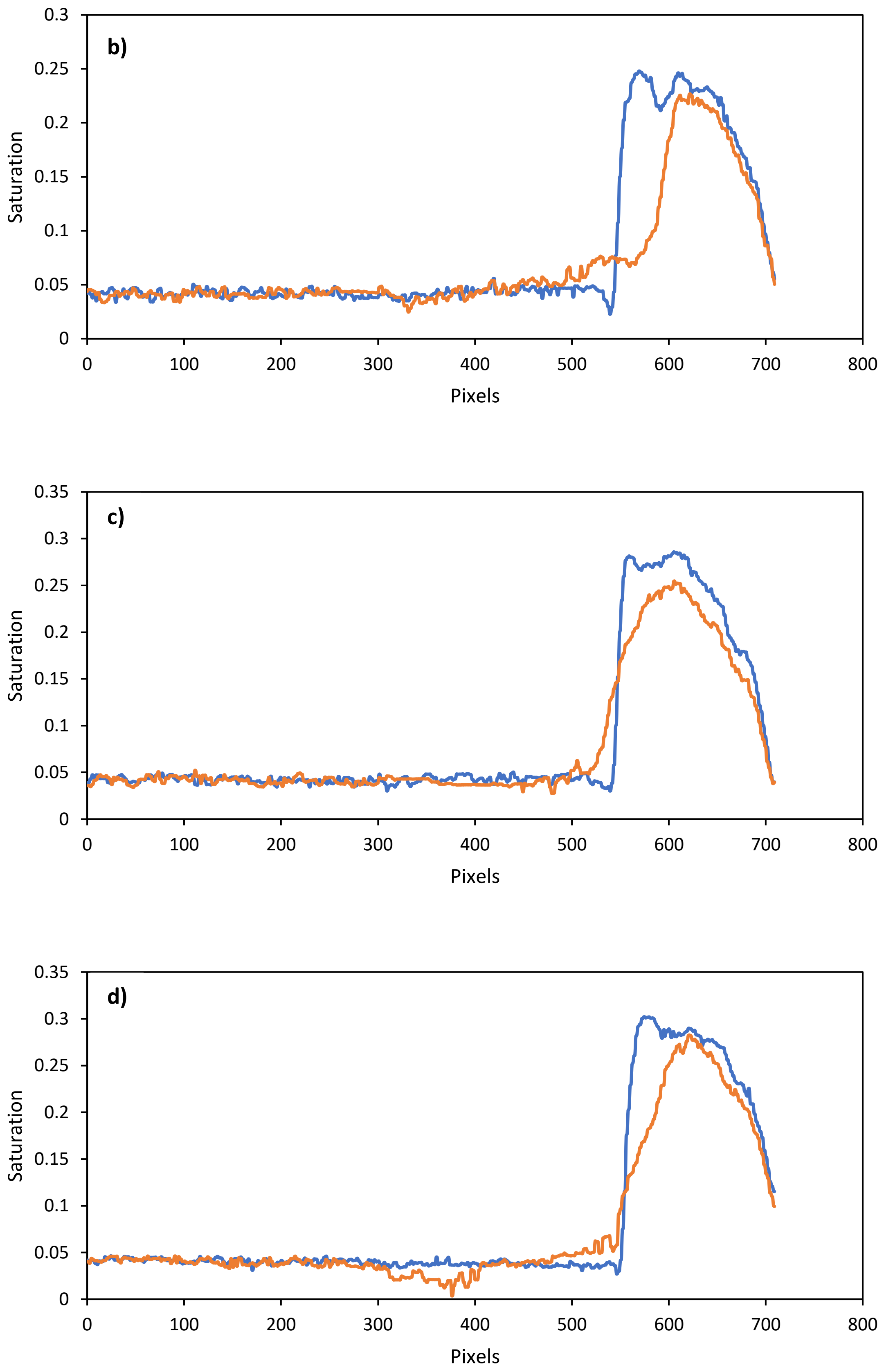

Fig. S4. Plot saturation profile through the paper strip (distance in pixels) before (blue) and after 60 seconds (orange) dropping buffer solution with **A**: 10, **B**: 25, **C**: 40, and **D**: 59 mg/mL of pullulan-based cocktails.

Table S2. Anthocyanins dragged from sensor zone with pullulan as retention polymers in saturation variation terms.

Pullulan concentration mg/mL	% Anthocyanins Dragged from sensor zone (difference between time 60 and 0 of Saturation) / Anthocyanins Retained
10	24.2% / 75.7%
25	24.2% / 75.7%
40	13.6% / 86.3%
50	14.8% / 85.1%

S7. Anthocyanins concentration optimization

The anthocyanins extract concentration in the cocktail was optimized to determine the most suitable amount that would achieve a higher variance within our desired pH range and ensure the most reproducible performance. Since anthocyanins are the chromoreactant responsible for producing the sensor signal, its concentration in the cocktail is one of the critical factors to be optimized. Low concentrations of anthocyanins extract may not result in sufficient colour changes that can be captured by the camera device. On the other hand, a high concentration could interfere with the microfluidic characteristics of the sensor and increase the time required to reach equilibrium in the reaction, consequently prolonging the measurement process. To optimize the anthocyanins extract concentration in the cocktail sensor solution, an assay was conducted to measure the H values within the pH range of 4 to 9, using different concentrations. Triplicates of the sensors were loaded with 5 µL of 10 mg/mL, 15 mg/mL, 20 mg/mL and 25mg/mL of anthocyanins extract in a solution containing 40 mg/mL of pullulan (Fig. S5). Each buffer solution at pH 4, 4.5, 5, 5.5, 6, 6.5, 7, 7.5, 8, 8.5, and 9 was measured, and images were captured at 60 seconds after spotting 2 µL of buffer onto each sensor. The H values were obtained using a circular ROI of 9768 pixels.

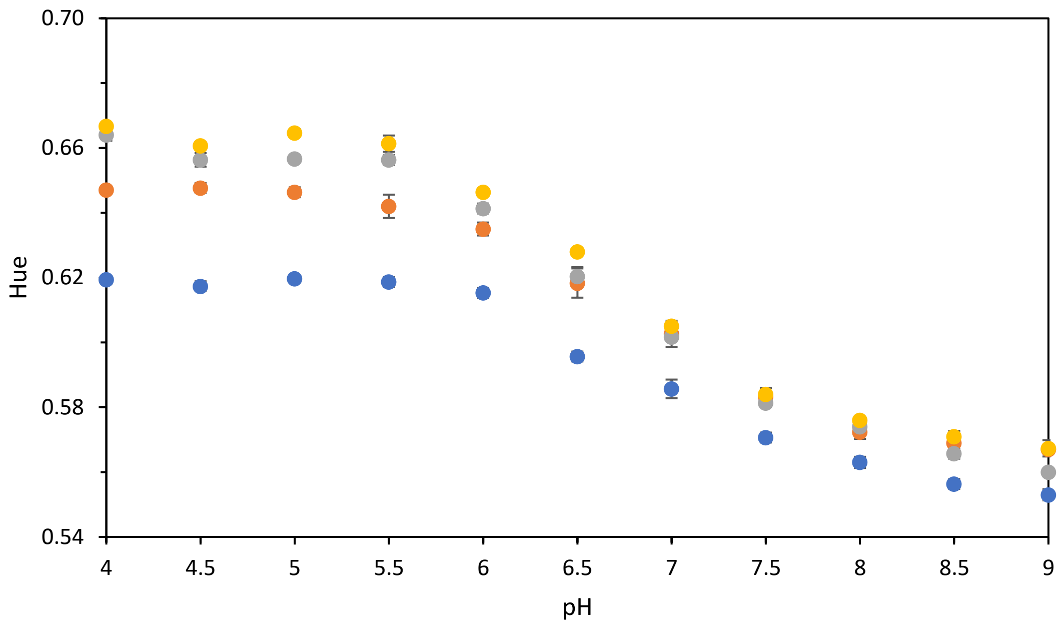

Fig. S5. Hue values of the pullulan-based cocktail at a concentration of 40 mg/mL with 10 (blue dots), 15 (orange dots), 20 (grey dots) and 25 mg/mL (yellow dots) of anthocyanins extract at different pH buffer solutions.

S8. Sensor cocktail volume optimization

The total volume of the cocktail mixture was also optimized. To achieve the highest variance in H values within the pH range of interest, specifically between pH 6 and 8, volumes of 3, 4, 5, and 6 µL of 0.5% (w/v) and 20 mg/mL of pullulan and anthocyanins extract, respectively, were tested (Table S3). For that purpose, triplicates were measured 60 seconds after spotting 2µL of pH 6 and 8 solutions.

Table S3. Volumes of cocktail loaded onto the paper-based sensor and their difference in H between pH 6 and 8.

Volume (µL)	ΔH
3	0.05
4	0.06
5	0.07
6	0.07

S9. Reaction Kinetics

The reaction time and the wetting capacity of the sensor were tested by loading 4 membranes with 40 mg/mL of pullulan cocktail and 10, 15, 20, and 25 mg/mL of anthocyanins extract concentration. Hue values were obtained after adding 2 µL of a buffer solution at pH 4 (Fig. S6). The same experiment was repeat with 5 µL of 0.5%

(w/v) concentration solution of chitosan and 20 mg/mL of anthocyanins extract (Fig. 3). Measurements were taken at various time intervals: from the moment the sample was spotted (time=0 s) up to 600 seconds, in intervals of 15 seconds between 0 and 60 s; 30 s between 90 and 180 s; and 60 between 240 and 600 s.

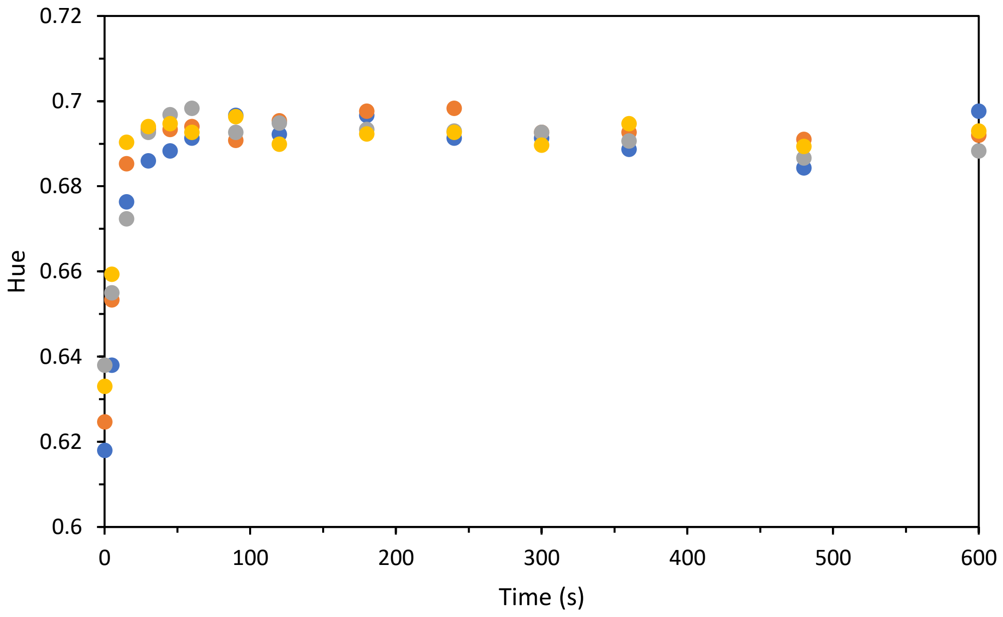

Fig. S6. Hue values of the pullulan-based cocktail at a concentration of 40 mg/mL with 10 (blue dots), 15 (orange dots), 20 (grey dots), and 25 (yellow dots) mg/mL of anthocyanins extract at different time from 0 to 600 s when the device was tested with pH 4.

S10. Calibration with camera setup

For the calibration of the sensor in the lightbox with the camera, 19 different pH solutions from pH 4 to pH 9 (4, 4.5, 5, 5.5, 6, 6.2, 6.4, 6.6, 6.8, 7, 7.2, 7.4, 7.6, 7.8, 8, 8.5 and 9) were used to measure the H value as analytical parameter 60 s after adding 2μL of each solution. The membranes were loaded with 5 μL of the optimized sensing cocktails containing either 40mg/mL of pullulan or 0.5% (w/v) of chitosan, along with 20 mg/mL of anthocyanins extract (Fig. S7). Subsequently, we allowed the membranes to dry overnight in a fridge at 4ºC following a 30-minute exposure in a stove at 50ºC. To capture the image, the camera was placed in a support focusing the inside of a lightbox where sensing membranes were placed. The digitalized images with the camera were analyzed using ImageJ software. The H values were obtained from a constant ROI of 9768 pixels after converting the image from RGB to HSV colour space. For this assay, we performed 3 replicates for each pH solution, and the average H values were fitted to a Boltzmann

equation (Equation S1) using Origin software. Precision was determined by calculating the average coefficient of variation (CV) of the results for each pH solution, considering all the replicates (Table S4).

$$y = A_2 + \frac{(A_1 - A_2)}{1+e^{\frac{(x-A_3)}{A_4}}} \qquad \textbf{(Equation S1)}$$

Table S4. Analytical parameter for polymers-based cocktail in light box.

Cocktail	A1	A2	x0	dx	R^2	Range	Precision
Pullulan	0.661	0.560	6.696	0.457	0.995	5.5-7.8	0.21%
Chitosan	0.712	0.571	6.559	0.397	0.997	5.4-7.8	0.60%

A)

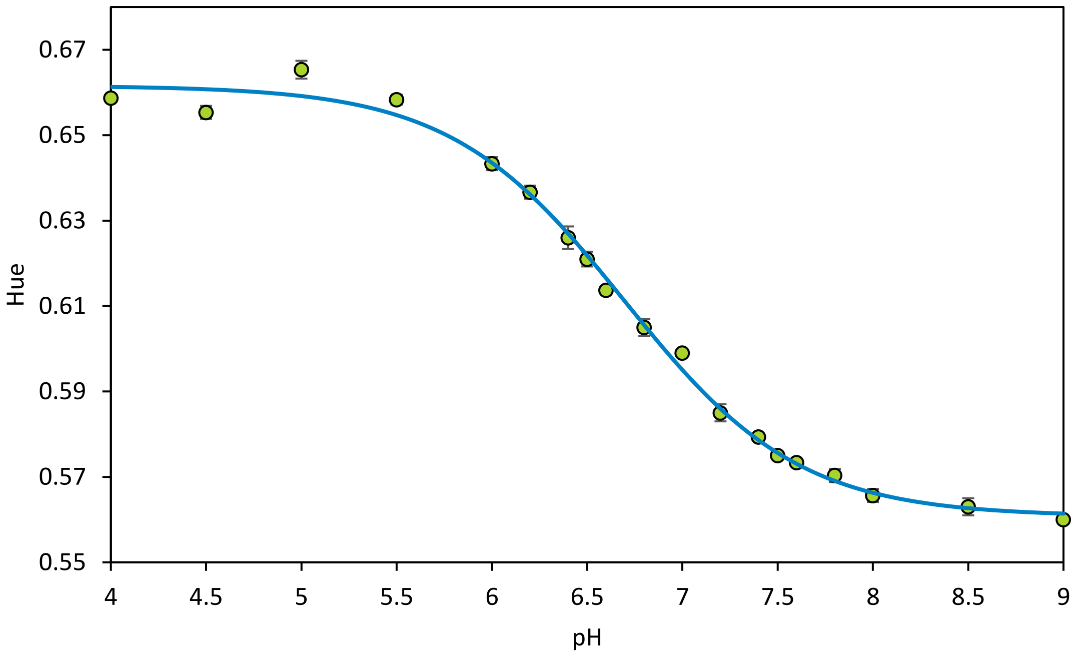

B)

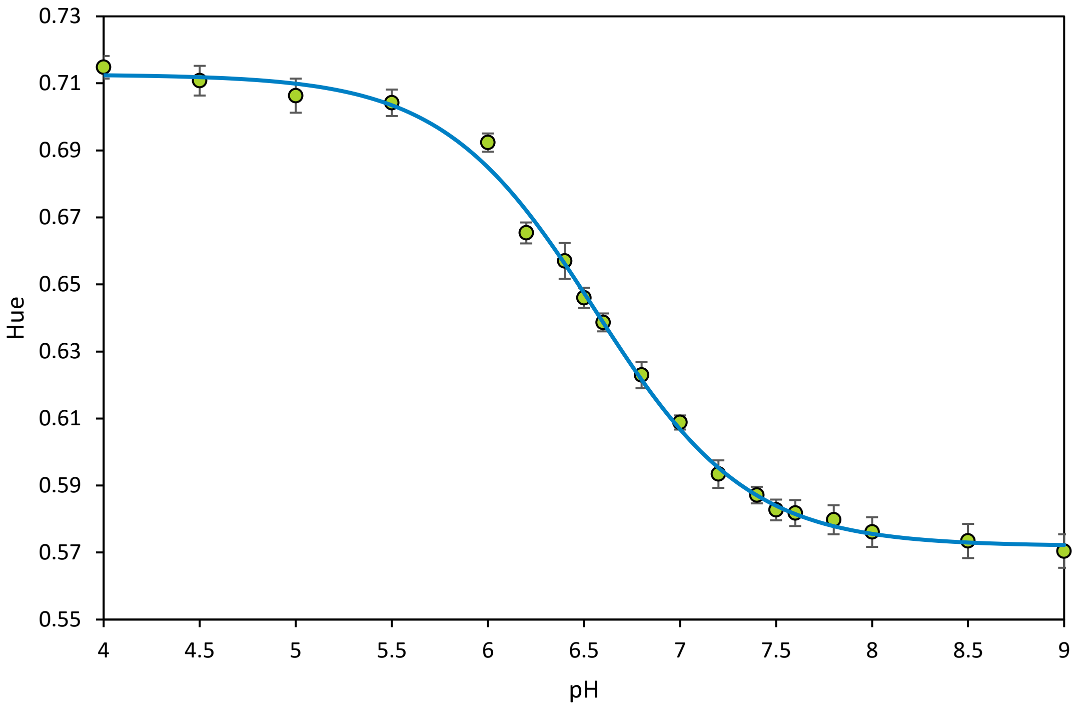

Fig. S7. A) Optimized pullulan-based cocktail membranes calibration. B) Optimized chitosan-based cocktail membranes calibration.

S11. Calibration with smartphone and custom app

The µPAD was analytically characterized using the developed smartphone-based platform with 13 different pH solutions ranging from pH 4 to 10 for the chitosan-based cocktail, and from pH 4 to 9 for the pullulan-based cocktail. In both cases, the H value was used as analytical parameter, measured 60 s after adding 2 µL of each solution. The membranes were loaded with 5 µL of chitosan-based cocktail containing 0.5% (w/v) and 20 mg/mL of chitosan and anthocyanins extract, respectively, and 5 µL of pullulan-based cocktail containing 40 mg/mL and 20 mg/mL of pullulan and anthocyanins extract, respectively (See Fig. 5). The loaded membranes were allowed to dry overnight in a refrigerator at 4ºC following a 30-minute incubation in a stove at 50ºC. For these calibrations, the sensing membranes were inserted into the disposable stick, which was designed to fit into the 3D-printed black accessory that attaches to the smartphone camera (Fig. 1). The smartphone camera, with the flash mode enabled, captured images using the custom-developed app. This app automatically processes the photographs to obtain RGB and HSV coordinates within a ROI adjusted to the sensing membrane. In this assay, three

replicates were used for each pH solution, and the average H values were fitted to a Boltzmann equation. Additionally, each membrane was measured three times by capturing three separate images to assess the technical reproducibility (Tech. Rep) of the entire assay using the developed system. This was achieved by calculating the average CV of each triplicate. Precision was determined by measuring the average CV of the results for each pH solution, considering all the replicates (Table S5).

Table S5. Analytical parameters obtained for the polymer-based cocktail using the smartphone-based platform.

Cocktail	A1	A2	x0	dx	R^2	Range	Precision	Tech. Rep.
Pullulan	0.761	0.552	6.264	0.769	0.991	4.9-8.0	0.77%	0.47%
Chitosan	0.798	0.522	6.720	0.528	0.997	5.4-8.1	1.58%	0.85%

S12. Automatic image processing algorithm

This section describes the methodology employed for the automatic image acquisition and processing tasks. The algorithms developed for this purpose are based on the open-source computer vision OpenCV 3.1.0 Android library, which is also available for the iOS platform and the Android OS, so the developed algorithms are potentially applicable to iOS smartphones and tablets. The image processing pipeline is initiated automatically by the app once the user loads or takes a photograph and clicks on the 'Process' button on the screen. The pipeline is designed to locate the circle-shaped sensor and compute the pH value from the detected colour. To enhance the reliability of the processing and eliminate unnecessary background, the app initially discards the parts of the image outside the area of interest. The resulting masked image or region of interest (ROI) is processed using the Circle Hough Transform (CHT) feature extraction technique provided by the OpenCV library, which detects circular objects in imperfect digital image inputs. Prior to using the circles' detection algorithms, a pre-processing stage is conducted to prepare the image for a successful CHT detection.

To prepare the image for successful Circle Hough Transform (CHT) detection, various pre-processing steps are applied. Firstly, the image is denoised using the *fastNlMeansDenoisingColoured()* function, which is a modified version of the *fastNlMeansDenoising()* function specifically designed for coloured images. This function utilizes the CIELAB colour space to separately denoise the *L* and *AB* components

based on the given *h* parameters. It is worth noting that the choice of *h* values can have an impact on the amount of noise and image details that are retained. In our implementation, the specific argument values used for all implemented functions are detailed in Table S6. Following denoising, the image is converted to grayscale, as subsequent functions require a grayscale image as input. This is accomplished using the *cvtColour()* method to convert the image from one colour space to another. A thresholding operation is then applied, as it was experimentally determined that this step significantly improved the reliability and accuracy of the subsequent circle detection algorithm. After testing various thresholding methods, it was concluded that a simple binary threshold operation was the most appropriate for our specific case. After the thresholding operation, the image is inverted using the *Core.bitwise_not()* function. Finally, a blur filter is applied to reduce noise and prevent false detections. Image blurring is accomplished by convolving the image with a low-pass filter kernel, which is commonly used to eliminate high-frequency noise. In our implementation, the simple *blur()* function was utilized with a normalized box filter to blur the thresholded image.

Table S6. Android functions and their corresponding arguments used for the image processing tasks in the custom-developed smartphone application.

Function	Argument	Explanation	Value
fastNlMeans DenoisingColoured ()	*templateWindowSize*	Size in pixels of the template patch that is used to compute weights	7
	searchWindowSize	Size in pixels of the window that is used to compute weighted average for given pixel	21
	h	Parameter regulating filter strength for luminance component	10
	hColour	The same as *h* argument but for colour components	10
cvtColour()	*code*	Colour space conversion code	COLOUR _RGBA2GRA Y
Threshold()	*maxVal*	Maximum value which is assigned to pixel values exceeding the threshold	255
	thresh	Threshold value	235
	type	Type of thresholding operation	THRESH _BINARY
blur()	*ksize*	Blurring kernel size	5x5
	method	Detection method	CV_HOUGH _GRADIENT
	dp	Inverse ratio of the accumulator resolution to the image resolution	1.2

	minDist	Minimum distance between the centers of the detected circles	2000
	param1	First method-specific parameter. In case of HOUGH_GRADIENT, it is the higher threshold of the two passed to the Canny edge detector	10
	param2	Second method-specific parameter. In case of HOUGH_GRADIENT, it is the accumulator threshold for the circle centers at the detection stage	35
	minRadius	Minimum circle radius	45
	maxRadius	Maximum circle radius	65

Following the blurring operation, the Hough Transform function of OpenCV was employed to identify circles in the photograph of the pH sensing device. The *HoughCircles()* function was utilized, which is based on the mathematical representation of a spot/circle as $(x - x_c)^2 + (y - y_c)^2 = r^2$, where (x_c, y_c) is the center of the circle and r is its radius. The Hough Gradient method was utilized by the OpenCV HoughCircles transform, which leverages the gradient information of edges to improve efficiency in terms of memory requirements and processing speed. First, the image was passed through a Canny edge detector, which significantly reduces the amount of data that needs to be processed. Subsequently, the local gradient was computed for all the non-zero points in the result image from the edge detector using the Sobel operator to calculate the x- and y-derivatives. Next, every point along the line that the slope indicates was incremented in a two-dimensional accumulator, taking into consideration a specified minimum and maximum distance. Concurrently, the algorithm stored the location of every one of these non-zero pixels. The centers of the candidate detected spots were then selected from the points in the accumulator, provided that these points were above a given threshold and larger than their neighbors. The candidate centers were then arranged in descending order according to their accumulator values. Subsequently, all the non-zero points for each candidate center were sorted based on their distance to this center. Finally, a center was chosen if it had sufficient support from the non-zero points around it, and it was far enough from any previous center. To implement the Hough Transform function, several critical parameters must be specified as arguments, and their chosen values are detailed in Table S6. The *param1* and *param2* parameters are noteworthy among them, which are the edge threshold used by the Canny edge detector and the accumulator threshold, respectively. In this regard, *param1* determines the sensitivity, setting how strong the spot edges must be, while *param2* establishes the amount of edge points required to declare

that a circle has been detected. Therefore, a compromise must be made between these two parameters to detect the circle-shaped sensor accurately. The optimal values were obtained experimentally for the correct identification of the pH sensor. Additionally, the app generates a plain text report that is stored in the phone's memory and contains information about the detected sensor and its colour coordinates in the RGB and HSV colour spaces. The results, including the image and report, can be shared through various messaging and cloud services such as Facebook, Twitter, WhatsApp, or email, among others.

S13. Stability performance

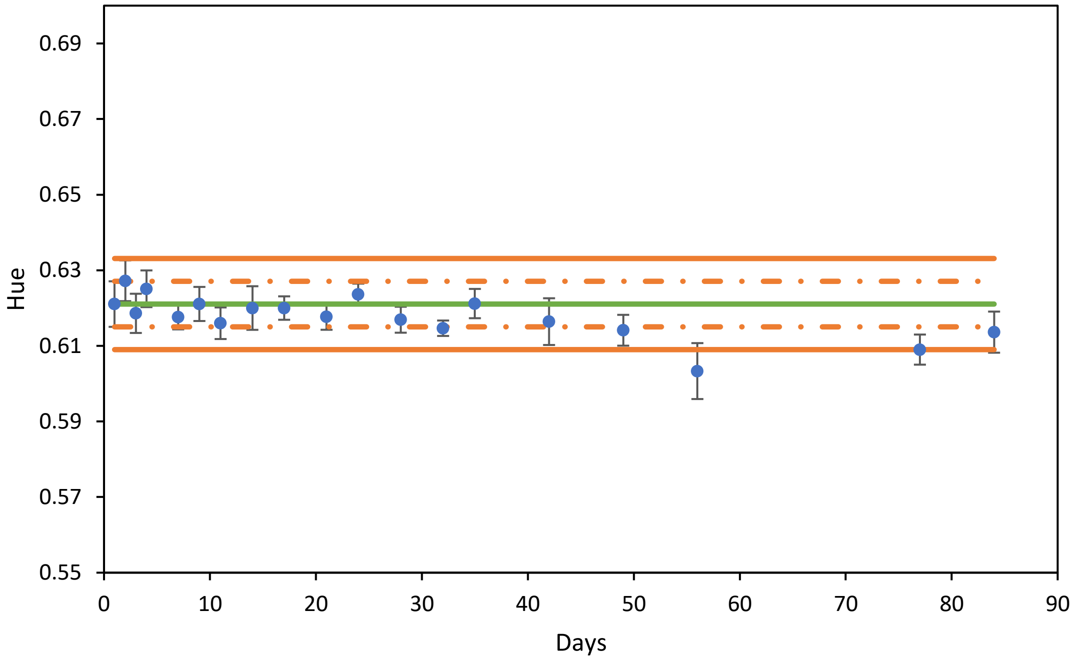

Fig. S8. Stability assay chart where green line is the main reference H value from day 1. Orange discontinuous lines represent the ±σ range and the orange continuous lines represent the ±2σ values from the main reference H value.

S14. SOP for sample collection and individual exclusion

Exclusion criteria for individuals for sample collection:

- Being under twenty years old.
- Having an infection or buccal disease.
- Having diabetes.
- Having eaten or drunk less than two hours prior to the sample collection.

- Having brushed teeth less than two hours before sample collection.

Exclusion criteria for saliva collection:

- Samples containing sputum or mucus.
- Sample containing blood.

Sample collection procedure:

1. Mouth rinse with water (without drinking it) 10 min before the sample collection.
2. The disposable stick was placed on the tongue, ensuring contact with any surface with saliva using the hole on the back of the stick.
3. Remove when the sensing membrane on its upper face becomes wet.

S15. Validation of polymers-based cocktail with smartphone app

Table S7. Chitosan-based cocktail validation with smartphone app.

Sample	pH meter	Chitosan based device	|Error|%
1	7.22	7.51	4.0
2	7.35	7.43	1.2
3	7.15	7.04	1.5
4	7.47	7.20	3.4
6	7.18	7.22	0.6
7	7.45	7.20	3.3
8	7.20	7.00	2.7
9	7.19	6.96	3.0
10	7.30	7.12	2.3
11	7.15	6.88	3.7
12	7.40	7.47	1.0
13	7.31	7.38	0.9
14	7.29	6.95	4.6
15	7.13	7.18	0.7
16	7.26	7.28	0.3
17	7.28	7.15	1.7
18	7.33	6.95	5.1

Table S8. Pullulan-based cocktail validation with smartphone app.

Sample	pH meter	Pullulan Based device	|Error|%
1	7.19	7.73	7.5
2	7.12	7.91	11.2
3	7.22	8.47	17.3
4	6.87	7.62	10.9
5	7.16	7.50	4.9
6	6.95	6.86	1.2

7	7.08	8.05	13.8
8	7.30	7.73	5.9
9	7.00	9.06	29.5
10	7.20	8.19	13.8
11	7.32	8.85	20.9
12	6.64	6.53	1.6
13	6.96	7.63	9.7
14	7.24	7.24	0.1

S16. 3D-printed accessories

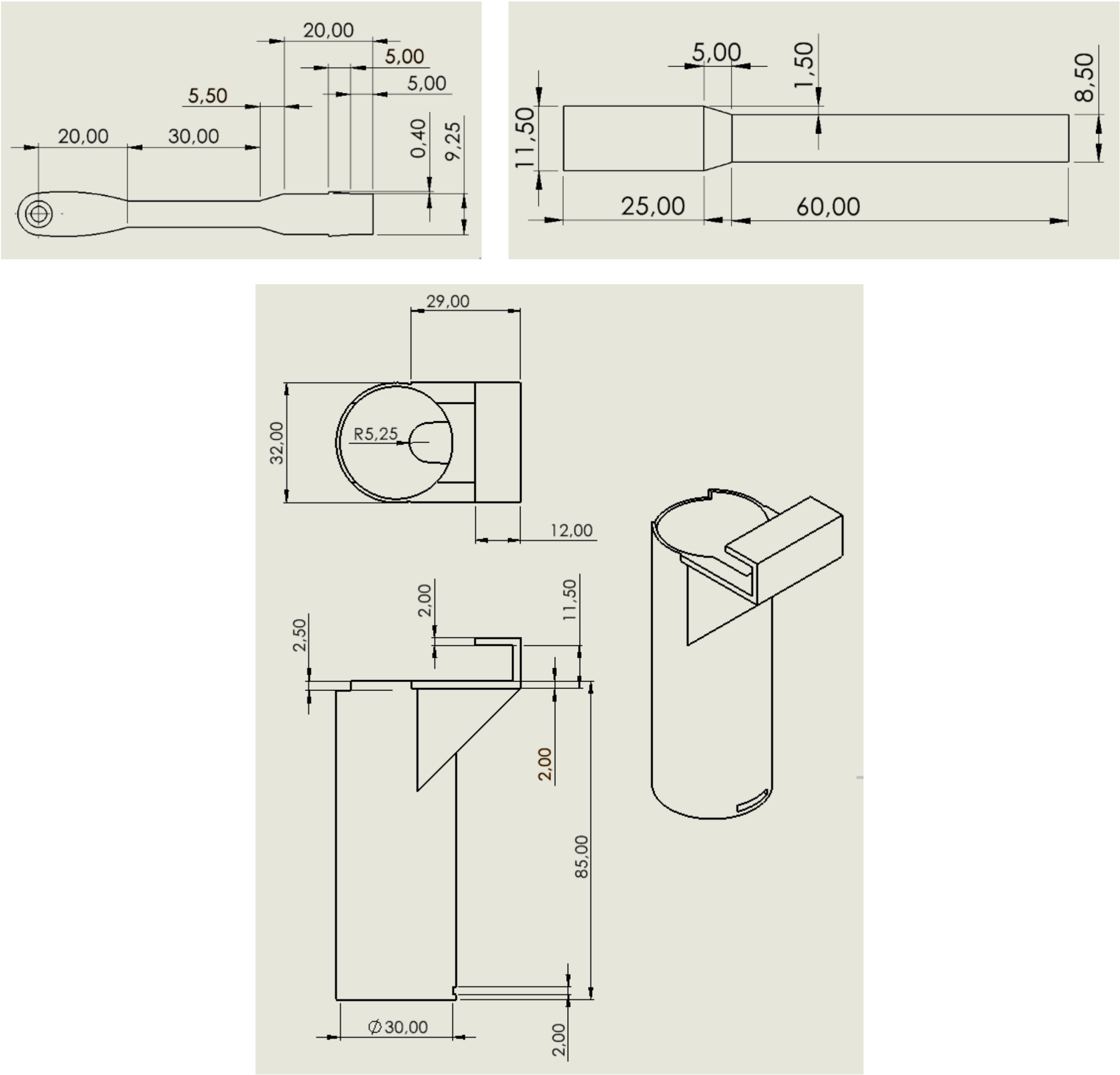

Fig. S9. Design drawings with dimensions in mm of the customized 3D-printed accessories.
